# Supporting Health Care Professionals to Improve the Processes of Shared Decision Making and Self-Management in a Web-Based Intervention: Randomized Controlled Trial

**DOI:** 10.2196/jmir.3170

**Published:** 2014-10-21

**Authors:** Barbara Sassen, Gerjo Kok, Jan Schepers, Luc Vanhees

**Affiliations:** ^1^Faculty of Health CareInnovation in Health CareUniversity of Applied SciencesUtrechtNetherlands; ^2^Health SciencesWork and Social PsychologyMaastricht UniversityMaastrichtNetherlands; ^3^Health SciencesFaculty of Psychology and NeurosciencesMaastricht UniversityMaastrichtNetherlands; ^4^RehabilitationRehabilitation SciencesKU LeuvenLeuvenBelgium

**Keywords:** Web-based intervention, health professionals, RCT, self-management, barriers

## Abstract

**Background:**

Research to assess the effect of interventions to improve the processes of shared decision making and self-management directed at health care professionals is limited. Using the protocol of Intervention Mapping, a Web-based intervention directed at health care professionals was developed to complement and optimize health services in patient-centered care.

**Objective:**

The objective of the Web-based intervention was to increase health care professionals’ intention and encouraging behavior toward patient self-management, following cardiovascular risk management guidelines.

**Methods:**

A randomized controlled trial was used to assess the effect of a theory-based intervention, using a pre-test and post-test design. The intervention website consisted of a module to help improve professionals’ behavior, a module to increase patients’ intention and risk-reduction behavior toward cardiovascular risk, and a parallel module with a support system for the health care professionals. Health care professionals (n=69) were recruited online and randomly allocated to the intervention group (n=26) or (waiting list) control group (n=43), and invited their patients to participate. The outcome was improved professional behavior toward health education, and was self-assessed through questionnaires based on the Theory of Planned Behavior. Social-cognitive determinants, intention and behavior were measured pre-intervention and at 1-year follow-up.

**Results:**

The module to improve professionals’ behavior was used by 45% (19/42) of the health care professionals in the intervention group. The module to support the health professional in encouraging behavior toward patients was used by 48% (20/42). The module to improve patients’ risk-reduction behavior was provided to 44% (24/54) of patients. In 1 of every 5 patients, the guideline for cardiovascular risk management was used. The Web-based intervention was poorly used. In the intervention group, no differences in social-cognitive determinants, intention and behavior were found for health care professionals, compared with the control group. We narrowed the intervention group and no significant differences were found in intention and behavior, except for barriers. Results showed a significant overall difference in barriers between the intervention and the control group (*F*_1_=4.128, *P*=.02).

**Conclusions:**

The intervention was used by less than half of the participants and did not improve health care professionals’ and patients’ cardiovascular risk-reduction behavior. The website was not used intensively because of time and organizational constraints. Professionals in the intervention group experienced higher levels of barriers to encouraging patients, than professionals in the control group. No improvements were detected in the processes of shared decision making and patient self-management. Although participant education level was relatively high and the intervention was pre-tested, it is possible that the way the information was presented could be the reason for low participation and high dropout. Further research embedded in professionals’ regular consultations with patients is required with specific emphasis on the processes of dissemination and implementation of innovations in patient-centered care.

**Trial Registration:**

Netherlands Trial Register Number (NTR): NTR2584; http://www.trialregister.nl/trialreg/admin/rctview.asp?TC=2584 (Archived by WebCite at http://www.webcitation.org/6STirC66r).

## Introduction

In health care, the focus is on optimizing patient self-management. Patients should manage their own health with the support of health care professionals. For targeted and effective self-management, shared decision making is a prerequisite. Shared decision making to improve self-management is more than offering professional support or increasing knowledge about patients’ health problem(s). In patient-centered care, patients and health care professionals should cooperate, exchange their own relevant information, and work together optimizing self-management to achieve intended outcomes. It results in better patient outcomes when health care professionals encourage their patients to be involved in decision making. A review showed that professionals tend to misjudge patients’ ability to be involved in decision making [1]. Shared decision making is not broadly implemented by health care professionals in clinical practice, and the intention of professionals to engage in or use interventions to facilitate shared decision making is suboptimal [1,2].

To facilitate shared decision making with the objective of optimizing self-management, interventions directed at the health care professional is an option to explore. Intervention Mapping provides a framework to develop systematically planned, theory- and evidence-based interventions [3-6]. Intervention Mapping is used throughout the process of creating an intervention, from diagnosis of the problem to problem solution, and includes collaborating iteratively with priority groups, stakeholders, and experts in the fields of health education and health promotion. Intervention Mapping consists of six planning steps in which each step has a different task and is a prerequisite for the next step. The intervention development process should start by assessing the social-cognitive determinants of the behavior under study. This is followed by choosing and applying methods to change these determinants and behavior. Intervention Mapping places specific emphasis on the transparency of the translation of evidence-based behavior change techniques in intervention components. This is to develop the intervention, explain its rationale, and to facilitate replication [4]. The outcome measures of the intervention should include behavior as well as determinants that influence the behavior [3]. Intervention Mapping has been found to be effective for developing interventions with the objective of changing the behavior of health care professionals and patients, and has led to interventions with effects on patients’ behavior [3,7-11].

Information and communication technologies (ICT) in the health care domain (eHealth) can facilitate communication and improve the health of patients and the quality of health care [12,13]. eHealth used as a clinical decision support tool for health care professionals has this potential and can improve clinical practice, though nonusage attrition is a documented problem [12,14,15]. The acceptance of eHealth depends on health care professionals’ perception of its usefulness (with an impact on intention), next to the perceived ease of use, and the facilitating and inhibiting conditions, as described in the Technology Acceptance Model [16]. Decision support systems improve clinical practice when these are provided automatically as part of the workflow, in time and at location, and when recommendations are provided [15]. A systematic review showed that computer-based clinical decision support systems can enhance health care professionals’ delivered preventive care [17]. In a meta-analysis it was concluded that Web-based instruction for health care professionals had positive effects [18]. But a review of the effectiveness of interventions to promote adoption of ICT showed that there is limited evidence of effective interventions for health care professionals [12]. For patients, Web-based interventions have been effective in boosting health-related changes and improving their health behaviors, though maintenance of the behavior change is often a problem [19]. A meta-analysis of behavioral change outcomes showed that Web-based interventions are effective in achieving changes in knowledge and in the behavior of patients [20]. Web-based interventions that are theory-based and use multiple behavior change techniques, and especially when based on the Theory of Planned Behavior, can change patients’ behavior [21,22].

Research to assess the effects of interventions to improve the processes of shared decision making and self-management directed at health care professionals is limited [1]. We hypothesized that in the clinical practice of patient-centered care, shared decision making can optimize self-management using an eHealth-application. In this paper, we report on the results of a randomized controlled trial regarding the implementation of a Web-based intervention directed at health care professionals. The objective of the Web-based intervention was to increase health care professionals’ intention and encouraging behavior toward patient self-management, following cardiovascular risk management guidelines [23]. In this paper, we followed the Consolidated Standards of Reporting Trials (CONSORT) criteria [24,25].

## Methods

### Participants

Participants were health care professionals with at least a bachelor’s degree in nursing or physiotherapy and who had regular consultations with patients with cardiovascular risk factors (ie, abdominal obesity, high blood pressure, low high-density lipoprotein cholesterol, elevated triglycerides, and elevated blood glucose levels) and low levels of physical activity [26-29]. The health care professionals were former students of the Faculty of Health Care, University of Applied Sciences (Netherlands) and were invited to participate via a personalized email. All participants were offered a 3-hour training session to work with the website and 12 participants attended the training. Participants could choose not to attend the training and instead use the online instruction tool. Much effort was put in motivating the participants in the intervention group to use and keep using the website. For follow-up contact, we used emails and telephone contact.

### Study Design

**Figure 1:**
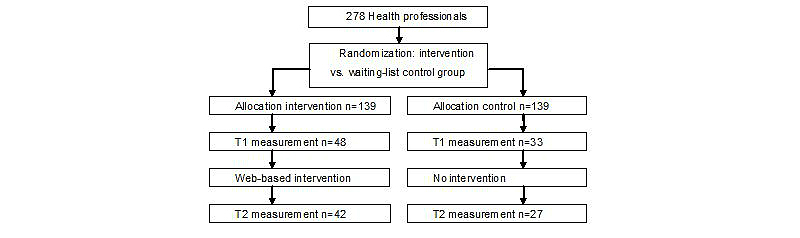
Intervention flow chart.

A randomized controlled trial was performed using a pre-test (T1) and post-test (T2) design, to determine the effectiveness of a clinical decision support system used to optimize shared decision making and the self-management of patients. Health care professionals were the unit of randomization and were randomly allocated to the intervention versus the waiting list control group. Health care professionals invited their patients. The recommendation was to use the intervention for every patient that fit the intervention. Patients were informed about the study and gave informed consent. The study sample size was based on the outcome of improved professionals’ behavior toward patient-centered care. Power analysis estimated how many respondents were needed for the study to find a significant difference in health care professionals’ behavior. This analysis (power 0.80; alpha=.05, two-tailed) revealed that 62 professionals in each condition were needed. Randomization was based on a random number sequence, using a computer randomized number generator. The total group of 278 professionals was randomized and drop by drop assigned to the intervention versus the control group; 81 professionals were willing to participate (Figure 1). The professionals in the waiting list control group did not receive further intervention, until after T2. The professionals in the control group did not receive a log-in code, and did not invite or enlist patients. A total of 12 professionals were “lost”, 9 for not using their log-in code and 3 of them changed their email accounts. Data collection via the website took place at T1 and T2 after 12 months. Initially the follow-up period was 6 months; however, due to low participation rates, we extended the follow-up period. Professionals used the website from January 2011 till June 2012.

Outcomes were self-assessed through a questionnaire based on the Theory of Planned Behavior [30,31]. The questionnaire was part of the website and used at T1 and T2. The content was derived from a literature review and in-depth interviews with health care professionals on how they encourage patients. There were eight elicitation interviews held, four with health care professionals with a background in physiotherapy and four with a background in nursing. Four of those professionals were observed in their professional activities for a regular working day. For measuring social-cognitive determinants, no valid questionnaires are available, but only valid procedures. The construction of the questionnaire is according to the Theory of Planned Behavior, specific to the definition of the behavior and the specification of the research population [4,31]. The questionnaire was piloted and as a result no revisions were made. Answers ranged on a 7-point scale (1=“definitely not” to 7=“most definitely”).

We assessed *“*behavior” with two items: “Do you encourage cardiovascular patients to increase their physical activity?”, and “How often do you encourage cardiovascular patients to become physically active?” (Cronbach alpha=.64). “Intention” was indexed with three questions: “Do you intend to encourage cardiovascular patients to become physically active tomorrow and the day after tomorrow?”, “Do you expect to encourage cardiovascular patients to become physically active tomorrow and the day after tomorrow?”, and “Of the first 10 cardiovascular patients you see, how many do you intend to encourage to become physically active?” (Cronbach alpha=.82). *“*Attitude” was assessed by: “In my view, encouraging cardiovascular patients to become physically active is very good - very bad” and “Encouraging cardiovascular patients is very useful - very useless”. Then we asked, “Is it useful to: assess patients’ motivation, assess the pros and cons of physical activity, teach patients how to resist social pressure, teach patients specific skills pertaining to physical activity, teach patients how to handle barriers in regard to physical activity, formulate physical activity goals together with patients, teach patients how to handle relapses, and help patients understand the relationship between the specific health problem and physical inactivity?”. These eight items (“Is it useful to…”) were averaged and that score was averaged with the first two item scores to represent attitude (Cronbach alpha=.63).

“Perceived behavioral control” (PBC) was assessed by: “Do you think that you have the skills and knowledge to encourage cardiovascular patients to become physically active?”, “Do you think you can rely on your skills and knowledge to encourage cardiovascular patients to become physically active?”, and third we asked, “Encouraging every cardiovascular patient to become physically active is very difficult (1) - very easy (7)”. PBC was further assessed by eight items that paralleled the eight items used for attitudes: “It is very difficult (1) - very easy (7) to: assess patients’ motivation, assess the pros and cons of physical activity, teach patients how to resist social pressure, teach patients specific skills pertaining to physical activity, teach patients how to handle barriers in regard to physical activity, formulate physical activity goals together with patients, teach patients how to handle relapses, and help patients understand the relationship between the specific health problem and physical inactivity?”. Once again, this scale score was calculated and combined with the previous three items as a measure of PBC (Cronbach alpha=.68). “Subjective norm” was measured by four items: “Most colleagues who are important to me think I should encourage cardiovascular patients to become physically active”, “Most colleagues value that I encourage cardiovascular patients to become physically active”, “Patients value that I encourage them to become physically active”, and “The organization I work for values that I encourage cardiovascular patients to become physically active” (Cronbach alpha=.73). *“*Moral norm” was assessed by three questions: “Encouraging patients to engage in physical activity is…my professional duty, …a moral obligation, and …an obvious part of my job” (Cronbach alpha=.76). A determinant score was calculated for every social-cognitive determinant. *“*Habit” was measured by two questions: “Encouraging patients to be physically active is something I do without thinking, and …something I do automatically” (Cronbach alpha=.75). “Barriers” were indexed by two questions that focused on encouraging patients “even when one is busy” and “even when one’s organization makes it difficult to encourage patients” (Cronbach alpha=.69).

### Intervention

**Figure 2:**
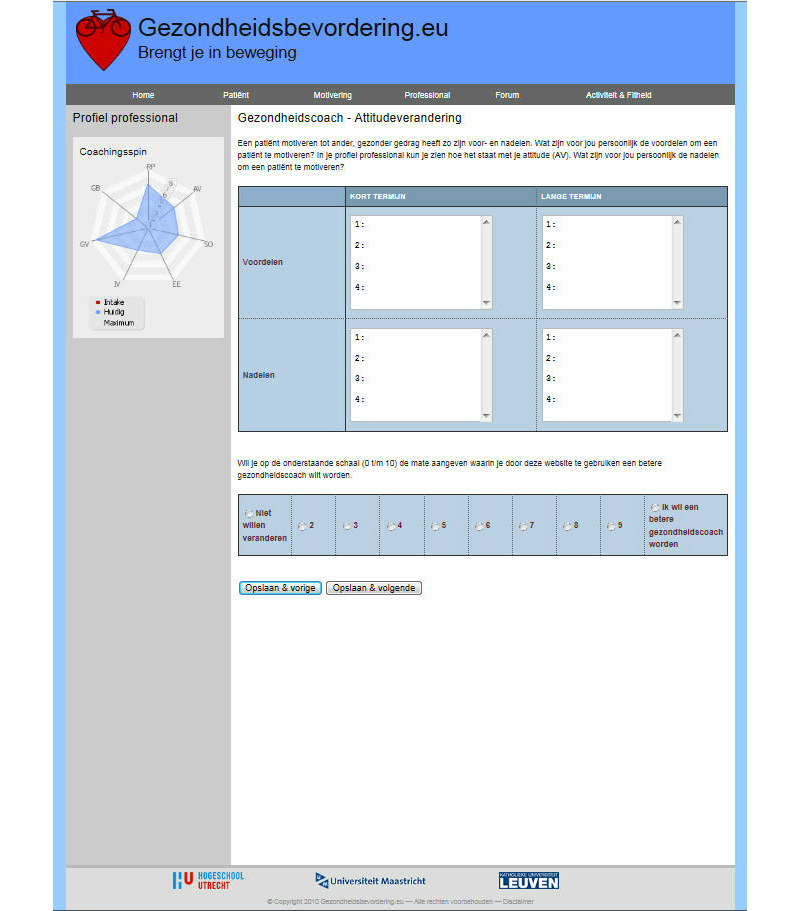
Intervention screenshot.

**Figure 3:**
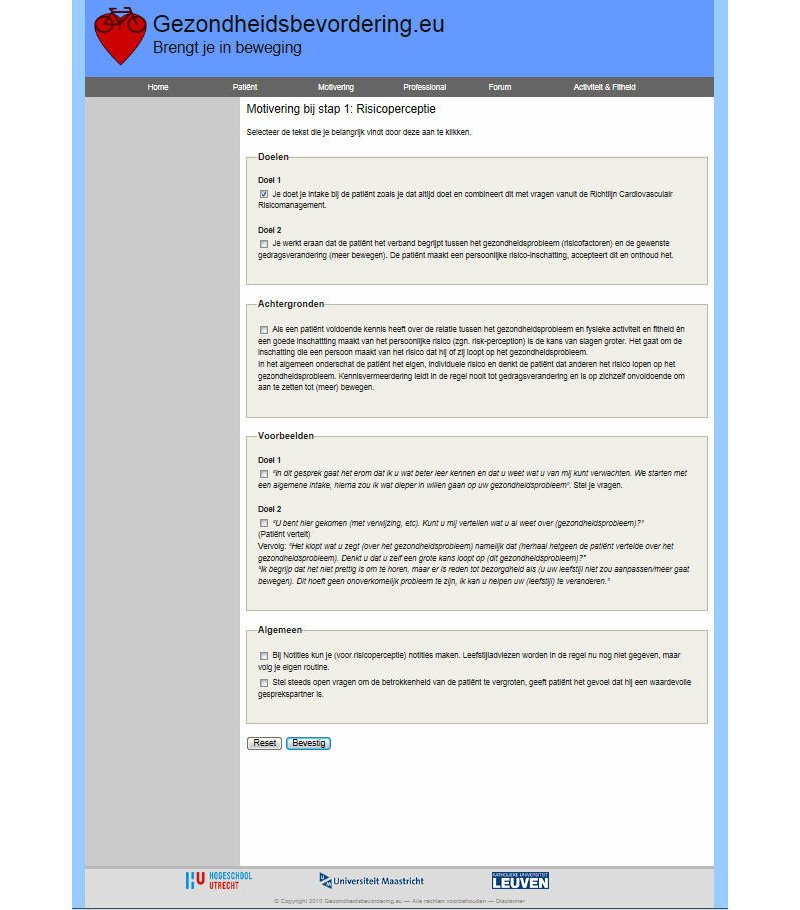
Intervention screenshot.

**Figure 4:**
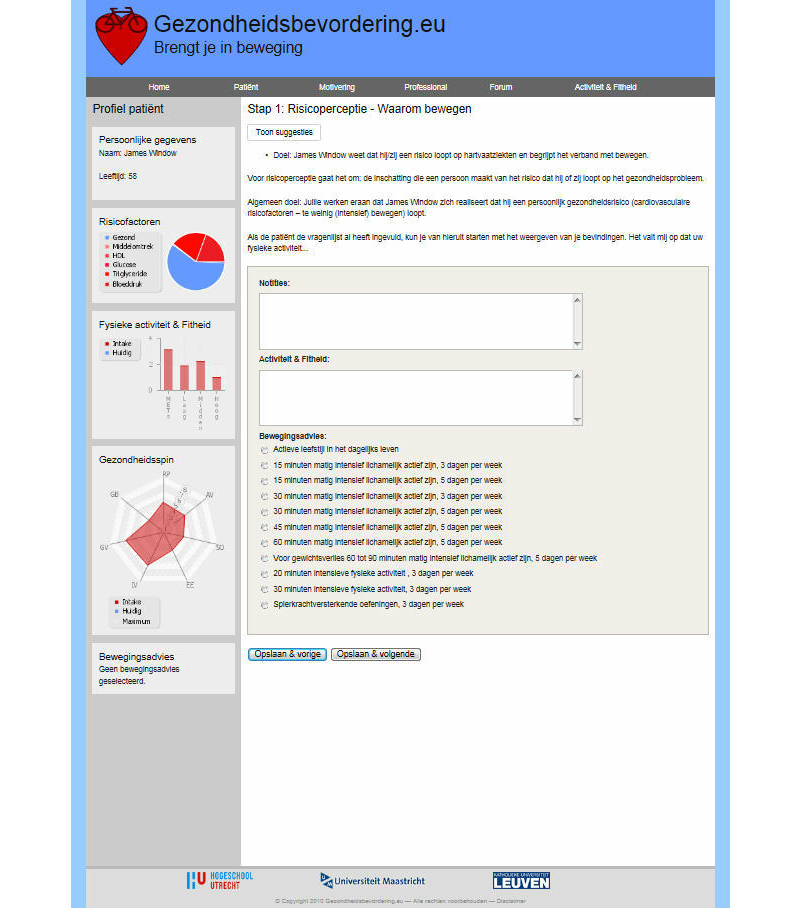
Intervention screenshot.

Participants had access to the website [32], which offered several modules. The development and content of the intervention is described in detail elsewhere [7,30]. Modules and a forum were directed at the health care professional to increase professionals’ awareness of their thoughts, and learn skills and strategies to support patients in their own self-management, this to improve their intention and behavior toward patient-centered health education. The first module enclosed a set of seven screens to help the professional to improve his or her professional behavior (Figure 2). The screens contained self-complete forms and were designed and pre-tested to educate the health care professional, with a personal feedback system in a “coaching spider chart”. The screens started with “risk” communication to support thinking about encouraging patients. This was followed by listing the pros and cons of encouraging patients in the short- and long-term. Hereafter, the health professional was encouraged to seek support and look at the sub-skills needed to be an encouraging health professional. Next, there was a screen for planning the encouraging behavior change, making a plan, and putting the behavior change into practice. The identification of high-risk situations and the practice of coping responses were encouraged. To enhance effective patient-centered health education, the website also included a second module with a support system for the health professional, parallel to the module for encouraging the patient (Figure 3). The third module consisted of a maximum of seven consultations to encourage the patient with cardiovascular risk factors, easily adaptable to the needs and individual characteristics of the patients (Figure 4). This module started with risk perception to encourage the patient to think about individual cardiovascular risk and personal vulnerability, followed by encouraging the patient to describe what the personal pros and cons are (not) to becoming physically active in the short- and long-term. With the support of the professional, the patient was encouraged to recognize social pressure, seek social support, and practice sub-skills. The patient was supported in planning the behavior and putting it into practice, detecting high-risk situations, and practicing coping responses. The third module started with the assessment following cardiovascular risk management guidelines. The screens contained the patient’s profile with a feedback system on the progression in behavior change in a spider chart, physical activity levels in bar graphs, and cardiovascular risk factors in a pie chart. The website helped the patient to look back at the plans made in conjunction with the health care professional. The website provided a fourth module with specific information on physical activity devices, planning physical activity, and cardiovascular risk factors. The website also included a link to a forum directed at health care professionals to share experiences with other professionals in the intervention group. The intervention was extensively tested, but not piloted. The website underwent no changes during implementation. Institutional affiliations were displayed on the website. The study was approved by the Research Ethics Board at Maastricht University and was registered in the Dutch Trial Register (Trial ID: ECP-92, NTR2584).

### Statistical Analysis

Descriptive statistics were calculated and chi-square analyses were used to characterize the study groups at baseline and to determine the use of the website. We used paired *t* tests to evaluate the differences at T1 and T2, for the intervention group and the control group. Subsequently, we applied the method of General Linear Modeling with repeated measures to explore the overall change between the intervention and control group. Because we noticed from the process evaluation data that 16 health care professionals in the intervention group did not start using the website, we applied the method of General Linear Modeling with repeated measures again; for this analysis, these 16 professionals were transferred to the waiting list control group. IBM SPSS version 20.0 was applied and a *P* value of ≤.05 was considered as statistically significant.

## Results

Table 1 shows the baseline demographics of the study participants in the intervention and waiting list control group. A total of 69 health care professionals were identified, with 42 professionals participating in the intervention group: 69% (29/42) female, mean age 38.6 years (SD 11.3); 79% bachelor’s degree, 21% higher education; with mean 9.76 years (SD 8.5) of professional experience; 44% worked as a soloist or with 1 or 2 colleagues, and 56% worked together with at least 3 others). Professionals in the intervention group stated that 59% of their consultation time was devoted to health education. No significant differences between the intervention group and the control group (n=27) were found at baseline (T1).

**Figure 5:**
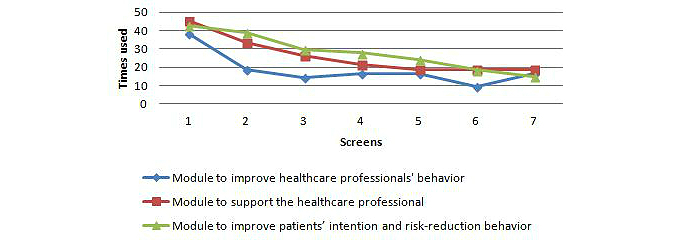
Use of the intervention website modules.

Figure 5 provides a representation of the use of different modules on the website: a module to improve professional behavior, a module to support the health professional, and a module to improve patients’ intention and risk-reduction behavior. The module to improve the professionals’ behavior to optimize processes of shared decision making and self-management, was used by 45% of the professionals (19/42). Of the professionals in the intervention group, 19% (8/42) used only one of seven screens; 7% (3/42) used all seven screens. The screen to support thinking about encouraging patients was used by 38% (16/42) of the professionals. In 19% (8/42) of the cases, pros and cons of encouraging patients in the short- and long-term were listed, 14% (6/42) used the screen to seek support, and 17% (7/42) looked at the sub-skills needed. In addition, the screen for planning the encouraging behavior was used by 17% (7/42) of the professionals, for putting the behavior change into practice by 10% (4/42), and for maintaining the encouraging behavior, 17% (7/42).

The module with background information on how to coach the patient with the aim of supporting the health professional in his or her encouraging behavior toward patients was used by 48% (20/42) of the professionals; 17% (7/42) of the professionals used all seven screens; 45% (19/42) used the screen how to encourage a patient to think about his/her personal risk; 33% (14/42) used the screen how to list pros and cons with a patient; 26% (11/42) used the screen how to seek support; and 21% (9/42) how to practice the sub-skills needed. The last screens in this module (planning the encouraging behavior, putting the behavior change into practice, and maintaining the behavior) were used by 19% (8/42) of the health care professionals. The forum directed at improving social support was used by 4 health care professionals.

Health care professionals invited 54 patients in the intervention: 56% (30/54) male, mean age 50.9 years (SD 11.8), with differing educational degrees. Health care professionals assessed cardiovascular risk and 19% (10/54) of the patients had two or more cardiovascular risk factors, and/or a heart disease and/or diabetes; 13% (7/54) were physically active for at least 30 minutes, for 5 days per week. In 82% (44/54) of the patients, the guidelines to assess cardiovascular risk were not used by the health professional. The module to improve patients’ intention and risk-reduction behavior, with the purpose of increasing the processes of shared decision making and self-management, was provided to 44% (24/54) of the patients by a health professional (Figure 2). Of the professionals that used this module together with their patients, 43% (23/54) provided their patient(s) with risk-perception. A total of 39% (21/54) provided attitudinal change and outcome expectations, 30% (16/54) resistance to social pressure and seek support, and 28% (15/54) provided encouraging sub-skill enactment. For 24% (13/54) of the patients, the professional provided a planning of the behavior change; 19% (10/54) provided putting the behavior change into practice; and 15% (8/54) provided maintaining the behavior change.

Table 2 shows the baseline score at T1 and T2, for the intervention group and the control group. At measurement T1, the professionals in the intervention group had positive intentions (mean 6.25, SD 1.00) and positive attitudes (mean 6.30, SD 0.44) to encouraging patients with cardiovascular risk factors, but, in comparison, the mean scores on the self-reported behavior (mean 4.54, SD 1.02), perceived behavioral control (mean 4.65, SD 0.79), and subjective norms (mean 5.48, SD 0.55) were modest. At T1, the mean intention score was 6.25 (SD 1.00) and the behavior score was 4.54 (SD 1.02); at T2, the mean intention score was 6.06 (SD 1.11) and behavior score was 4.63 (SD 0.85). In the intervention group, no differences in social-cognitive determinants, intention and behavior were found when we compared T1 and T2, except for a difference in perceived behavioral control (*t*_26_=−2.954, *P*=.00, effect size=0.50). In the control group, also no differences were detected between the measurement at T1 compared with T2; but, contrary to expectations, we detected an increase in perceived behavioral control (*t*_19_=−2.651, *P*=.02, effect size=0.54). When we compared the intervention group with the professionals in the control group, no significant differences between the intervention and control group were found in social-cognitive determinants, intention and behavior; the detected difference in the intervention and control group in perceived behavioral control turned out to be overall non-significant when we compared the intervention with the control group.

When we narrowed the intervention group (n=26) by transferring the professionals who did not use the website to the waiting list control group (n=43), no significant differences between the intervention and control group were found in social-cognitive determinants, intention and behavior, except for perceived behavior control and barriers (Table 3). There was a difference in perceived behavioral control in the intervention group (*t*_19_=−2.485, *P*=.02, effect size=−0.30), and also in the control group (*t*_18_=−3.105, *P*=.00, effect size=−0.23). This detected difference in perceived behavioral control turned out to be overall non-significant. Results showed a significant overall difference in barriers between the intervention and the control group (*F*_1_=4.128, *P*=.02). Professionals in the intervention group experienced higher levels of barriers to encouraging patients, than professionals in the control group.

**Table 1:** Baseline demographics of study participants *(*N*=*69).

	Intervention group (n=42) n (%)	Control group (n=27) n (%)	*P* value
Gender, female	29 (69%)	21 (78%)	*.*428
Age, years, mean (SD)	38.6 (11.3)	39.7 (8.4)	.062
Education, bachelor’s degree	26 (79%)	13 (68%)	.406
Education, degree above bachelor’s	7 (21%)	6 (32%)	N/A
Professional experience, years, mean (SD)	9.76 (8.5)	9.58 (9.1)	.910
Working as a soloist, or with 1 or 2 colleagues	16 (44%)	5 (21%)	.060
Working with 3 or more colleagues	20 (56%)	19 (79%)	N/A
Consultation time devoted to health education	36 (59%)	23 (54%)	.508

**Table 2:** Paired differences between intervention group and control group, measured at T1 and T2.^a^

		Intervention group (n=42) mean (SD)	Control group (n=27) mean (SD)	*F* test
**Behavior**				
	T1	4.54 (1.02)	4.83 (0.69)	*P*=.68
	T2	4.63 (0.85)	4.79 (0.82)	
**Intention**				
	T1	6.25 (1.00)	5.87 (1.15)	*P*=.12
	T2	6.06 (1.11)	6.02 (0.91)	
**Attitude**				
	T1	6.30 (0.44)	6.23 (0.69)	*P*=.64
	T2	6.30 (0.56)	6.31 (0.68)	
**Perceived behavioral control** ^b^				
	T1	4.65 (0.79)	4.90 (0.87)	*P*=.98
	T2	5.04 (0.73)	5.28 (0.80)	
**Subjective norms**				
	T1	5.48 (0.55)	5.58 (0.93)	*P*=.64
	T2	5.57 (0.63)	5.74 (0.76)	
**Moral norms**				
	T1	6.04 (0.63)	6.20 (0.59)	*P*=.75
	T2	6.19 (0.70)	6.30 (0.55)	
**Barriers**				
	T1	3.11 (1.17)	2.78 (1.01)	*P*=.46
	T2	3.18 (1.12)	2.63 (0.96)	

^a^social-cognitive variables range 1-7.

^b^Intervention group: Cohen’s *d*=−1.15, effect size=0.50; Control group: Cohen’s *d*=−1.28, effect size= 0.54.

**Table 3:** Differences between narrowed intervention group and control group, measured at T1 and T2.^a^

		Intervention group (n=26) mean (SD)	Control group (n=43) mean (SD)	*F* test
**Behavior**				
	T1	4.38 (1.04)	4.91 (0.67)	*P*=.76
	T2	4.45 (0.81)	4.89 (0.81)	
**Intention**				
	T1	6.10 (1.06)	6.08 (1.10)	*P*=.33
	T2	5.93 (1.21)	6.13 (0.85)	
**Attitude**				
	T1	6.28 (0.47)	6.26 (0.63)	*P*=.86
	T2	6.29 (0.56)	6.31 (0.64)	
**Perceived behavioral control** ^b^				
	T1	4.63 (0.82)	4.85 (0.83)	*P*=.96
	T2	5.01 (0.76)	5.23 (0.77)	
**Subjective norms**				
	T1	5.46 (0.59)	5.57 (0.84)	*P*=.91
	T2	5.60 (0.63)	5.68 (0.73)	
**Moral norms**				
	T1	5.88 (0.60)	6.28 (0.58)	*P*=.41
	T2	6.08 (0.73)	6.35 (0.53)	
**Barriers**				
	T1	3.09 (1.11)	2.78 (1.11)	*F*_*1*_ *=*4.128 *P*=.05
	T2	3.40 (1.09)	2.59 (0.94)	

^a^social-cognitive variables range 1-7.

^b^Intervention group: Cohen’s *d*=−0.63, effect size=−0.30; Control group: Cohen’s *d*=−0.47, effect size=−0.23.

## Discussion

### Principal Findings

In this paper, we report on the results of a randomized controlled trial testing the effectiveness of a Web-based intervention in the clinical practice of patient-centered care. The intervention was developed to optimize processes of shared decision making and self-management, following the protocol of Intervention Mapping. The objective was to increase health care professionals’ intention and behavior toward encouraging patient self-management.

Results indicated no intervention effect on the outcome measure of our study: the encouraging behavior of health care professionals. Results indicated no overall differences for social-cognitive determinants, intention and behavior, when the intervention group was compared with the control group. We narrowed the intervention group and took a closer look at the health care professionals that used the Web-based intervention. Results showed that these professionals experienced higher levels of barriers, meaning that time and organizational constraints withheld them and obstructed the planned behavior to encourage patients, compared with the professionals in the control group. Next to the overall results of the intervention, we took a closer look at possible effects in the (initial) intervention group. Results indicated a medium-size effect for perceived behavioral control, with no effect for the other social-cognitive determinants, intention and behavior. Professionals in the intervention group increased their perceived behavioral control and reported that they had more control over their skills necessary to encourage patients. The same effect was seen in the control group, which means that there was no overall effect when we compared the intervention group with the control group.

Our study showed that health care professionals had high intentions and planned their encouraging behavior. It also showed that health care professionals had positive attitudes and described more pros than cons toward encouraging patients. Further, the study showed a positive moral norm to be an encouraging professional. But scores on behavior were modest in comparison, and though health care professionals did plan the encouraging behavior, they did not practice the encouraging behavior. Also scores on subjective norm (meaning that colleagues, patients, and the organization value their encouragement) and scores on perceived behavior control as the skills needed, were modest. Attendance on the Web-based intervention and use of the website was sub-optimal. Less than half of the health care professionals used the module to change their professional behavior, and/or used the module to get support in their encouraging behavior toward patients, and/or used the module to improve patients’ intention and risk-reduction behavior. The module to improve patients’ intention and risk-reduction behavior was used most, followed by the module to support the health professional. The module to change professional behavior was the least used. Only in 1 of every 5 patients was the guideline following cardiovascular risk management used. We hypothesized that in the clinical practice of patient-centered care, shared decision making can optimize self-management using an eHealth-application, but we were not able to detect improvements in the processes of shared decision making and self-management of the patient.

Systematic reviews showed a clear relationship between the intentions of health care professionals and their subsequent behavior; these were found to be appropriate to predict their behavior, and can be used to improve behavior change interventions targeting health care professionals [33,34]. Although the intention to employ interventions to facilitate shared decision making is often suboptimal, the health care professionals in our study showed high intention scores [1,2]. If intention scores are already high at baseline, it is difficult to change the behavior scores in a positive direction. Research showed that medium-to-large changes in intention scores are needed to show small-to-medium changes in behavior [35]. That improvement in intention was not sufficient to change the behavior, is often a problem and can be related to the fact that the effectiveness of interventions is reduced with increasing levels of standard care [36,37]. We detected an increase in perceived behavioral control and other research showed that health care professionals’ perceived behavior control is an important determinant of behavior to improve shared decision making [1]. The improvement in perceived behavioral control is important, because this can lead to progress in (planning) the encouraging behavior. But it is unclear if we can attribute the change in perceived behavioral control as an intervention effect, because we also detected an increase in the control group. In our study, health care professionals experienced barriers that hinder them in their encouraging behavior, meaning that time and organizational constraints obstruct them in improving the processes of shared decision making and self-management in patient-centered care. Another intervention directed at optimizing shared decision making also showed that lack of time was a barrier although intentions were high [1,38]. Other research showed that barriers for using an intervention are also that health care professionals do not use the applications, poor usability or integration into professionals’ workflow, non-acceptance of recommendations, and also the intervention’s inapplicability due to patient characteristics and the clinical situation [1,17,38]. In our study, changing the health behavior in line with evidence-based recommendations as described in the guideline for cardiovascular risk management proved difficult, but was similar with other studies where only small or no effects were found [39].

The application of evidence-based behavior change techniques used in our intervention should offer insight regarding how an intervention may change intention and behavior. When intention and perceived behavioral control are targeted in an intervention, clinician behavior can be improved [35]. Methods used in our Web-based intervention were action planning and coping planning; however, better results on intention and the (maintenance) of the behavior change were not reached [36]. Professionals’ perception of perceived behavioral control is an important determinant of behavior to improve shared decision making [1]. The use of the method-guided practice with feedback probably did lead to increased perceived behavioral control and better skills. But, use of the method decisional balance to encourage listing of pros and cons of changing the behavior did not lead to better results on attitudes. The use of the method resistance to social pressure and mobilizing others for social support, showed a slight but non-significant increase in subjective norm. We showed that professionals did not use or discontinued using the Web-based intervention. Though health care professionals stated that they spend a lot of time on health education (58.4% in the intervention group and 54.4% in the control group), and the pre-test before implementation of the intervention showed that a more evidence-based and systematic approach could be done in the same time, health care professionals lost interest in the intervention and stopped using the application. This nonuse of the intervention or nonusage attrition is a documented problem in the search for intervention effects [14]. Eysenbach [14] called this the methodological challenge in the evaluation of eHealth applications. A clinical decision support system can improve health care professionals’ performance when users are automatically prompted to use the website, but in our study the professionals themselves had to initiate use of the system [17]. A factor that may have influenced performance and attrition may be a lack of (immediate) advantage of working with the website for the health care professionals, or even encountering obstructions when working with the website. Another factor that may have influenced performance and attrition may be the compatibility with usual care and with workload. Also, the complexity of the intervention with (too) many modules may have influenced performance and attrition. The Web-based intervention was carefully developed following the Intervention Mapping protocol [7]. Health care professionals from the target group were involved in the development of the intervention, and they also pre-tested the application, but it may be that (too) much attention was directed at “does the website work as intended”.

### Limitations

In a review by Légare, it was concluded that sufficient enrollment of health care professionals and patients is often a problem and needs attention in research designs [1]. Our estimated sample size was not achieved, although recruitment and follow-up period was extended, and strong attempts were made to encourage professionals. Changing intentions and the encouraging behavior of health care professionals proved difficult with many inhibiting factors. An explanation is probably a ceiling effect for intention and attitude, so little progress can be expected in the intervention group. This combined with the fact that our measures were based on self-reported intention and behavior can have caused recall bias. Also, a possible explanation is a selection of professionals with special interest in health education, participating in our study. Although randomization ensured that participants were evenly distributed, we noticed that a few professionals were trained for another intervention but were not selected and they choose to attend our intervention. A limitation that might also have influenced outcomes is that the website had to be used during regular consultation with the patient. Though professionals stated that they spend much of their consultation time on health education, it was not just a matter of focus on self-management during consultation time. Another limitation that might influence the outcome is that the professionals needed more training on how to work with the modules of the Web-based intervention.

An online intervention can support health care professionals but training should be an important part of implementation. A total of 12 health care professionals attended a demonstration meeting, including professionals in the waiting list control group. It may be that training on the job can improve the use of the Web-based intervention. Training may increase professionals’ perception of perceived behavioral control, because professionals need to learn to use the specific clinical decision support tool [1,12,40]. Training that uses practice exercises, repetition, and feedback leads to improved learning outcomes for health care professionals [18,39]. Training may improve the process of shared decision making and self-management, as may the implementation of patient-mediated interventions such as decision aids [38]. Other important facilitators for dissemination and implementation of innovations are increasing health care professionals’ motivation, and showing the intervention’s innovative impact on the clinical process and on patient outcomes [1,41,42].

### Conclusions

The intervention was used by less than half of the participants and did not improve health care professionals’ and patients’ cardiovascular risk-reduction behavior. Health care professionals did not use the website intensively because of time and organizational constraints. Professionals in the intervention group experienced higher levels of barriers to encouraging patients, than professionals in the control group. We were not able to detect improvements in the processes of shared decision making and patient self-management. Although participant education level was relatively high and the intervention was pre-tested, it is possible that the way the information was presented could be the reason for low participation and high dropout. Further research embedded in professionals’ regular consultations with patients is required with specific emphasis on the processes of dissemination and implementation of innovations in patient-centered care.

## Multimedia Appendix 1

CONSORT-EHEALTH checklist V1.6.2 [25].

## Abbreviations

CONSORT: Consolidated Standards of Reporting Trials
ICT: information and communication technology
PBC: perceived behavioral control
