# Supplementary material for: Supporting Health Care Professionals to Improve the Processes of Shared Decision Making and Self-Management in a Web-Based Intervention: Randomized Controlled Trial
Source: J Med Internet Res. 2014 Oct 21;16(10):e211. doi: 10.2196/jmir.3170 (PMC4259881; doi:10.2196/jmir.3170)
Supplement: Supplementary file 1 [file jmir_v16i10e211_app1.pdf]

CONSORT-EHEALTH Checklist V1.6.2 Report

(based on CONSORT-EHEALTH V1.6), available at [http://tinyurl.com/consort-ehealth-v1-6].

3170

Date completed

4/25/2014 11:01:14

by

Barbara Sassen

Results of a Randomized Controlled Trial: Can we support healthcare professionals to improve the processes of shared-decision making and self management in a Web-based intervention?

TITLE

1a-i) Identify the mode of delivery in the title

Web-based intervention

Title:Results of a Randomized Controlled Trial: Can we support healthcare professionals to improve the processes of shared-decision making and self management in a Web-based intervention?

1a-ii) Non-web-based components or important co-interventions in title

Web-based intervention with face-to-face components.

1a-iii) Primary condition or target group in the title

Healthcare professionals

Title:Results of a Randomized Controlled Trial: Can we support healthcare professionals to improve the processes of shared-decision making and self management in a Web-based intervention?

ABSTRACT

1b-i) Key features/functionalities/components of the intervention and comparator in the METHODS section of the ABSTRACT

Intervention Mapping
Theory of Planned behavior

The Web-based intervention consisted of modules, including individualized text messages, self-completion forms, and charts and tables.
The website employed:
- a module to facilitate the professional to extent his professional behavior;
- a module to improve patients’ intention and risk-reduction behavior towards cardiovascular risk;
- with parallel a module with a support system for the healthcare professional.

1b-ii) Level of human involvement in the METHODS section of the ABSTRACT

Health professionals

1b-iii) Open vs. closed, web-based (self-assessment) vs. face-to-face assessments in the METHODS section of the ABSTRACT

Healthcare professionals (N = 69) were online recruited and randomly allocated to the intervention group (N=26) and (waiting-list) control group (N=43).
Healthcare professionals invited their patients to participate.

The study was a closed trial with face-to-face components.

1b-iv) RESULTS section in abstract must contain use data

Healthcare professionals (N = 69) were online recruited and randomly allocated to the intervention group (N=26) and (waiting-list) control group (N=43).

1b-v) CONCLUSIONS/DISCUSSION in abstract for negative trials

Conclusions: Since attendance and use of the Web-based intervention was sub-optimal, we cannot conclude that the intervention is ineffective. Rather, the healthcare professionals were not able to extend their professional encouraging behavior and were withheld to use the website because of time and organizational constraints. Professionals in the intervention group experienced higher levels of barriers to encourage patients, than professionals in the control group. We were not able to detect improvements in the processes of shared-decision making and self-management of the patients. Further research embedded in professionals' regular consultations with patients is required with specific emphasis on the processes of dissemination and implementation of innovations in patient-centered care. Training-on-the-job may improve eHealth use in healthcare settings (Trial ID: ECP-92, NTR2584).

Discussion

In this paper we report on the results of a randomized controlled trial testing the effectiveness of a Web-based intervention in the clinical practice of patient-centered care. The intervention was developed to optimize processes of shared-decision making and self management, following the protocol of Intervention Mapping. The objective was to extend the healthcare professionals' intention and behavior towards encouraging patients' self management.

Results indicated no intervention effect on the outcome measure of our study, the encouraging behavior of healthcare professionals. Results indicated no overall differences for social-cognitive determinants, intention and behavior, when the intervention group was compared with the control group. We narrowed the intervention group and took a closer look at the healthcare professionals that used the Web-based intervention. Results showed that these professionals experienced higher levels of barriers meaning that time and organizational constraints withheld them and obstruct the planned behavior to encourage patients, compared with the professionals in the control group. Next to the overall results of the intervention, we took a closer look at possible effects in the (initial) intervention group. Results indicated a medium size effect for perceived behavioral control, with no effect for the other social-cognitive determinants, intention and behavior. Professionals in the intervention group increased their perceived behavioral control and reported that they had more control over their skills necessary to encourage patients. The same effect was seen in the control group, which means that there was no overall effect when we compared the intervention group with the control group.

Our study showed that healthcare professionals had high intentions and are planning their encouraging behavior. Our study showed that healthcare professionals had positive attitudes and described more pros than cons towards encouraging patients. Our study also showed a positive moral norm to be an encouraging professional. But scores on behavior were modest in comparison, and though healthcare professionals are planning the encouraging behavior, they do not practice the encouraging behavior. Also scores on subjective norm meaning that colleagues, patients and the organization value their encouragement, and scores on perceived behavior control as the skills needed, were modest. Attendance to the Web-based intervention and use of the website was sub-optimal. Less than half of the healthcare professionals used the module to extend their professional behavior, and/or used the module to get supported in their encouraging behavior towards patients, and/or used the module to improve patients' intention and risk-reduction behavior. The module to improve patients' intention and risk-reduction behavior was used best, followed by the module to support the health professional. The module to extend professional behavior was the least used. Only in 1 of every 5 patients the guideline following cardiovascular risk management was used. We hypothesized that in the clinical practice of patient-centered care, shared-decision making can optimize self-management using an eHealth-application, but we were not able to detect clear improvements in the processes of shared-decision making and self-management of the patient.

Systematic reviews showed a clear relationship between the intentions of healthcare professionals and their subsequent behavior, these were found to be appropriate to predict their behavior, and can be used to improve behavior change interventions targeting healthcare professionals [32,33]. Although the intention to employ interventions to facilitate shared-decision making is often suboptimal, the healthcare professionals in our study showed high intention scores [1,2]. If intention scores are already high at baseline, as in our study, it is difficult to change the behavior scores in a positive direction. Research showed that medium-to-large changes in intention scores are needed to show small-to-medium changes in behavior [34]. That improvement in intention was not sufficient to change the behavior, is often a problem, and can be related to the fact that the effectiveness of interventions is reduced with increasing levels of standard care [35,36]. We detected an increase in perceived behavioral control and other research showed that healthcare professionals' perceived behavior control is an important determinant of behavior to improve shared-decision making [1]. The improvement in perceived behavioral control is important, because this can lead to progress in (planning) the encouraging behavior. But it is unclear if we can attribute the change in perceived behavioral control as an intervention effect, because we also detected an increase in the control group. In our study, healthcare professionals experienced barriers that hinder then in their encouraging behavior, meaning that time and organizational constraints obstruct them in improving the processes of shared decision-making and self management in patient centered care. Another intervention directed at optimizing shared decision-making, also showed that lack of time was a barrier although intentions were high [1,37]. Other research showed that barriers for using an intervention are also that healthcare professionals do not use the applications, poor usability or integration into professionals' workflow or non-acceptance of recommendations, and also the intervention's inapplicability due to patients characteristics and due to the clinical situation [1,37,17]. In our study, changing the health behavior in line with evidence-based recommendations as described in the guideline for cardiovascular risk management showed difficult; but similar with other studies where only small or no effects were found [38].

The application of evidence-based behaviour change techniques used in our intervention should offer insight regarding how an intervention may change intention and behaviour. When intention and perceived behavioral control are targeted in an intervention, clinician behavior can be improved [34]. Methods used in our Web-based intervention were action planning and coping planning, however better results on intention and the (maintenance) of the behavior change were not reached [35]. Professionals' perception of perceived behavioral control is an important determinant of behavior to improve shared-decision making [1]. The use of the method guided practice with feedback did probably lead to increased perceived behavioral control and better skills. But, use of the method decisional balance to encourage listing of pros and cons of changing the behavior, did not lead to better results on attitudes. The use of the method resistance to social pressure and mobilizing others for social support, showed a slight but non-significant increase in subjective norm. We showed that professionals did not use or discontinue using the Web-based intervention. Though healthcare professionals stated that they spend a lot of time on health education (58,4% in the intervention group and 54.4% in the control group), and the pretest showed that working more evidence-based and systematic could be done in the same time, healthcare professionals lost interest in the intervention and stopped using the application.This non-use of the intervention or nonusage attrition is a documented problem in the search for intervention effects [13]. Eysenbach [13] called this the methodological challenges in the evaluation of eHealth applications. A clinical decision support system can improve healthcare professionals' performance when users are automatically prompted to use the website; but in our study the professionals themselves had to activate the system making [17]. A factor that may have influenced performance and attrition may be a lack of (immediate) advantages of working with the website for the healthcare professionals, or even encountering obstructions when working with the website. Another factor that may have influenced performance and attrition may be the compatibility with usual care and with the workload. And also the complexity of the intervention with (to) many modules may have influenced performance and attrition. The Web-based intervention was carefully developed following the Intervention Mapping protocol [7]. Healthcare professionals from the target group were involved in the development of the intervention, and they also pretested the application, but maybe (to) much attention was directed at 'does the website work as intended'.

Limitations. In a review by Légare it was concluded that sufficient enrollment of healthcare professionals and patients is often a problem and needs attention in research designs [1]. Our estimated sample size was not achieved, although recruitment and follow-up period was extended, and strong attempts were made to encourage professionals. Changing intentions and the encouraging behavior of healthcare professionals and patients showed difficult with many inhibiting factors. An explanation is probably a ceiling effect for intention and attitude, so little progress can be expected in the intervention group. This combined with that our measures were based on self-reported intention and behavior that can have caused recall bias. Also, a possible explanation is a selection of professionals with special interest in health education, participating in our study. Although randomization made that participants were evenly distributed, we noticed that a few professionals were trained for another intervention but were not selected and they choose to attend our intervention. A limitation that might have influenced outcomes is that the website had to be applied during regular consultation with the patient. Though professionals stated that they spend much of their consultation time on health education, it showed not just a matter of focus on self

the patient. Though professionals stated that they spend much of their consultation time on health education, it showed not just a matter of focus on self management during consultation time. Another limitation that might influence the outcome is that the professionals needed more training how to work with the modules of the Web-based intervention.

An online intervention can support healthcare professionals but training should be an important part of implementation. A total of 12 healthcare professionals attended a demonstration meeting, including professionals in the waiting-list control group. Maybe training-on-the job can lead to better use of the Web-based intervention. Training may increase professionals' perception of perceived behavioral control, because professionals need to learn to use the specific clinical decision support tool [1,12]. Training that uses practice exercises, repetition and feedback leads to improved learning outcomes for healthcare professionals [18,38]. Training may improve the process of shared-decision making and self management, as may the implementation of patient-mediated interventions such as decision aids [37]. Important other facilitators for dissemination and implementation of innovations are increasing healthcare professionals' motivation, and showing the intervention's innovative impact on the clinical process and on patient outcomes [1,40,41]. Since attendance and use of the Web-based intervention was sub-optimal, we cannot conclude that the intervention is ineffective; rather, the healthcare professionals were not able to extend their professional encouraging behavior. Results showed that healthcare professionals experienced higher levels of barriers meaning that time and organizational constraints withheld them and obstruct the planned behavior to encourage patients, compared with the professionals in the control group. Further research embedded in professionals' regular consultations with patients is required with specific emphasis on the processes of dissemination and implementation of innovations in patient-centered care. Training-on-the-job may improve eHealth use in healthcare settings.

INTRODUCTION

2a-i) Problem and the type of system/solution

Research to assess the effect of interventions to improve the processes of shared-decision making and self management directed at healthcare professionals, is limited. Using the protocol of Intervention Mapping, a Web-based intervention directed at healthcare professionals was developed to complement and optimize health services in patient-centered care.

The objective of the Web-based intervention was to extent healthcare professionals' intention and encouraging behavior towards self management, following cardiovascular risk management guidelines.

2a-ii) Scientific background, rationale: What is known about the (type of) system

In health care, the focus is on optimizing the self management of patients. Patients should manage their own health with the support of healthcare professionals. For targeted and effective self management, shared-decision making is prerequisite. Shared decision making to improve self management is more than offering professional support or increasing knowledge about patients' health problem(s). In patient-centered care, patients and healthcare professionals should cooperate, exchange both their own relevant information, and work together optimizing self management to achieve intended outcomes. It results in better patient outcomes, when healthcare professionals encourage their patients to be involved in decision making. A review showed that professionals tend to misjudge patients to be involved in decision making [1]. Shared-decision making is not broadly implemented by healthcare professionals in clinical practice, and the intention of professionals to engage in or use interventions to facilitate shared-decision making is suboptimal [1,2].

To facilitate shared-decision making with the objective to optimize self management, interventions directed at the healthcare professional is an option to explore. Intervention Mapping provides a framework to develop systematically planned, theory- and evidence-based interventions [3,4,5,6]. Intervention Mapping is used throughout the process of creating an intervention, from diagnosis of the problem to problem solution, and includes collaborating iteratively with priority groups, stakeholders, and experts in the fields of health education and health promotion. Intervention Mapping consists of six planning steps in which each step has a different task and is a prerequisite for the next step The intervention development process should start by assessing the social-cognitive determinants of the behavior under study. This is followed by choosing and applying methods to change these determinants and behavior. Intervention Mapping places specific emphasis on the transparency of the translation of evidence-based behavior change techniques in intervention components. This is to develop the intervention, explain its rationale, and to facilitate replication [4]. The outcome measures of the intervention should include behavior as well as determinants that influence the behavior [3]. Intervention Mapping has been found to be effective for developing interventions with the objective to change the behavior of healthcare professionals and patients, and has led to interventions with effects on patients' behavior [3,7,8,9,10,11].

Information and communication technologies (ICT) in the healthcare domain (eHealth) can facilitate communication, and improve the health of patients and the quality of healthcare [12,14]. eHealth used as a clinical decision support tool for healthcare professionals has this potential and can improve clinical practice, though no usage attrition is a documented problem [12,13,15]. The acceptance of eHealth depends on healthcare professionals' perceived usefulness (with an impact on intention), next to the perceived ease of use, and the facilitating and inhibiting conditions, as described in the Technology Acceptance Model [16]. Decision support systems improved clinical practice when these are provided automatically as part of the workflow, in time and at location, and when recommendations are provided [15]. A systematic review showed that computer-based clinical decision support systems can enhance healthcare professionals' delivered preventive care [17]. In a meta-analysis it was concluded that internet-based instruction for healthcare professionals had positive effects [18]. But a review on the effectiveness of interventions to promote adoption of ICT, showed that there is limited evidence on effective interventions for healthcare professionals [12]. For patients, web-based interventions showed effective to boost health-related changes and improve their health behaviors, though maintenance of the behavior change is often a problem [19]. A meta-analysis of behavioral change outcomes showed that web-based interventions are effective in achieving changes in knowledge and in the behavior of patients [20]. Web-based interventions that are theory-based and use multiple behavior change techniques, and especially when based on the Theory of Planned Behavior, can change patients' behavior [21,22].

Research to assess the effects of interventions to improve the processes of shared-decision making and self management directed at healthcare professionals, is limited [1]. We hypothesized that in the clinical practice of patient-centered care, shared-decision making can optimize self-management using an eHealth-application. In this paper, we report on the results of a randomized controlled trial regarding the implementation of a Web-based intervention directed at healthcare professionals.

METHODS

3a) CONSORT: Description of trial design (such as parallel, factorial) including allocation ratio

The objective of the Web-based intervention was to extent healthcare professionals' intention and encouraging behavior towards self management, following cardiovascular risk management guidelines [23].

3b) CONSORT: Important changes to methods after trial commencement (such as eligibility criteria), with reasons

No changes were made

3b-i) Bug fixes, Downtimes, Content Changes

no changes were made during the implementation

4a) CONSORT: Eligibility criteria for participants

Participants were healthcare professionals with at least a Bachelor's degree in nursing or physiotherapy and who had regular consultations with patients with cardiovascular risk factors (i.e. abdominal obesity, high blood pressure, low high-density lipoprotein cholesterol, elevated triglycerides and elevated blood glucose levels) and low levels of physical activity [26,27,28,29]. The healthcare professionals were former students of the Faculty of Health Care, University of Applied Sciences and were invited to participate via a personalized email. All participants were offered a 3-hour training session to work with the website and 12 participants attended the training. Participants could choose not to attend the training and use the online instruction tool. Much effort was put in motivating the participants in the intervention group to use and keep using the website. For follow-up contact we used emails and telephone contact.

4a-i) Computer / Internet literacy

Participants were healthcare profesionals with at least a bachelor degree
4a-ii) Open vs. closed, web-based vs. face-to-face assessments:
Closed trial with face-to-face-components.

The healthcare professionals were former students of the Faculty of Health Care, University of Applied Sciences and were invited to participate via a personalized email.
4a-iii) Information giving during recruitment
All participants were offered a 3-hour training session to work with the website and 12 participants attended the training. Participants could choose not to attend the training and use the online instruction tool. Much effort was put in motivating the participants in the intervention group to use and keep using the website. For follow-up contact we used emails and telephone contact.
4b) CONSORT: Settings and locations where the data were collected
Participants were healthcare professionals ... who had regular consultations with patients with cardiovascular risk factors
4b-i) Report if outcomes were (self-)assessed through online questionnaires
Outcomes were self-assessed through a questionnaire based on the Theory of Planned Behaviour [30,31]. The questionnaire was part of the website and used at T1 and T2.
4b-ii) Report how institutional affiliations are displayed
Institutional affiliations (Maastricht university, Leuven University, University of Applied Sciences) were displayed on every screen.
5) CONSORT: Describe the interventions for each group with sufficient details to allow replication, including how and when they were actually administered
5-i) Mention names, credential, affiliations of the developers, sponsors, and owners
none
5-ii) Describe the history/development process
The development and content of the intervention is described in detail elsewhere:

Sassen B, Kok G, Mesters I, Crutzen R, Cremers A, Vanhees L. A web-based intervention for health professionals and patients to decrease cardiovascular risk attributable to physical inactivity: development process. JMIR Res Protoc. 2012 Dec 14;1(2):e21
5-iii) Revisions and updating
no changes were made
5-iv) Quality assurance methods
Cardiovascular risk management guidelines
5-v) Ensure replicability by publishing the source code, and/or providing screenshots/screen-capture video, and/or providing flowcharts of the algorithms used
The development and content of the intervention is described in detail elsewhere:

Sassen B, Kok G, Mesters I, Crutzen R, Cremers A, Vanhees L. A web-based intervention for health professionals and patients to decrease cardiovascular risk attributable to physical inactivity: development process. JMIR Res Protoc. 2012 Dec 14;1(2):e21
5-vi) Digital preservation
website www.gezondheidsbevordering.eu
5-vii) Access
login account
5-viii) Mode of delivery, features/functionalities/components of the intervention and comparator, and the theoretical framework
The development and content of the intervention is described in detail elsewhere:

Sassen B, Kok G, Mesters I, Crutzen R, Cremers A, Vanhees L. A web-based intervention for health professionals and patients to decrease cardiovascular risk attributable to physical inactivity: development process. JMIR Res Protoc. 2012 Dec 14;1(2):e21
5-ix) Describe use parameters
Use of the different modules on the website.

The Web-based intervention consisted of modules, including individualized text messages, self-completion forms, and charts and tables.
The website employed:
- a module to facilitate the professional to extent his professional behavior;
- a module to improve patients' intention and risk-reduction behavior towards cardiovascular risk;
- with parallel a module with a support system for the healthcare professional.

5-x) Clarify the level of human involvement
healthcare professionals
5-xi) Report any prompts/reminders used
All participants were offered a 3-hour training session to work with the website and 12 participants attended the training. Participants could choose not to attend the training and use the online instruction tool. Much effort was put in motivating the participants in the intervention group to use and keep using the website. For follow-up contact we used emails and telephone contact.
5-xii) Describe any co-interventions (incl. training/support)
All participants were offered a 3-hour training session to work with the website and 12 participants attended the training. Participants could choose not to attend the training and use the online instruction tool. Much effort was put in motivating the participants in the intervention group to use and keep using the website. For follow-up contact we used emails and telephone contact.
6a) CONSORT: Completely defined pre-specified primary and secondary outcome measures, including how and when they were assessed
The outcome was extended professional behavior towards health education. Outcomes were self-assessed through questionnaires based on the Theory of Planned Behaviour. Social-cognitive determinants, intention and behavior were measured pre-intervention and at one year follow-up.
6a-i) Online questionnaires: describe if they were validated for online use and apply CHERRIES items to describe how the questionnaires were designed/deployed
Outcomes were self-assessed through a questionnaire based on the Theory of Planned Behaviour [30,31]. The questionnaire was part of the website and used at T1 and T2. The content was derived from a literature review and in-depth interviews with healthcare professionals on how they encourage patients. There were eight elicitation interviews held, four with healthcare professionals with a background in physiotherapy and four with a background in nursing. Four of those professionals were observed in their professional activities for a regular working day. For measuring social-cognitive determinants, no valid questionnaires are available, but only valid procedures. The construction of the questionnaire is according to the Theory of Planned Behavior, specific to the definition of the behavior and the specification of the research population [4,31]. The questionnaire was piloted, as a result no revisions were made.

6a-ii) Describe whether and how “use” (including intensity of use/dosage) was defined/measured/monitored
Use of the modules, use of individualized text messages, self-completion forms, and charts and tables.
6a-iii) Describe whether, how, and when qualitative feedback from participants was obtained
Much effort was put in motivating the participants in the intervention group to use and keep using the website. For follow-up contact we used emails and telephone contact.
6b) CONSORT: Any changes to trial outcomes after the trial commenced, with reasons
-
7a) CONSORT: How sample size was determined
7a-i) Describe whether and how expected attrition was taken into account when calculating the sample size
The study sample size was based on the outcome of improved and extended professionals’ behavior towards patient-centered care. Power analysis estimated how many respondents were needed for the study to find a significant difference in healthcare professionals’ behavior. This analysis (power 0.80; alpha=0.05, two-tailed) revealed that 62 professionals in each condition were needed.
7b) CONSORT: When applicable, explanation of any interim analyses and stopping guidelines
-
8a) CONSORT: Method used to generate the random allocation sequence
Randomization was based on a random number sequence, using a computer randomized number generator.
8b) CONSORT: Type of randomisation; details of any restriction (such as blocking and block size)
The total group of 278 professionals was randomized and drop by drop assigned to the intervention vs. the control group; 81 professionals were willingly to participate (figure 1). The professionals in the waiting-list control group did not receive further intervention, until after T2.
9) CONSORT: Mechanism used to implement the random allocation sequence (such as sequentially numbered containers), describing any steps taken to conceal the sequence until interventions were assigned
Randomization was based on a random number sequence, using a computer randomized number generator.
10) CONSORT: Who generated the random allocation sequence, who enrolled participants, and who assigned participants to interventions
1 researcher
11a) CONSORT: Blinding - If done, who was blinded after assignment to interventions (for example, participants, care providers, those assessing outcomes) and how
11a-i) Specify who was blinded, and who wasn’t
The total group of 278 professionals was randomized and drop by drop assigned to the intervention vs. the control group; 81 professionals were willingly to participate (figure 1). The professionals in the waiting-list control group did not receive further intervention, until after T2. All participants were offered a 3-hour training session to work with the website and 12 participants attended the training.
11a-ii) Discuss e.g., whether participants knew which intervention was the “intervention of interest” and which one was the “comparator”
Healthcare professionals were the unit of randomization and were randomly allocated to the intervention vs. the waiting-list control group. The professionals in the waiting-list control group did not receive further intervention, until after T2. All participants were offered a 3-hour training session to work with the website and 12 participants attended the training. The healthcare professionals in the waiting-list control group were 'just' waiting
11b) CONSORT: If relevant, description of the similarity of interventions
-
12a) CONSORT: Statistical methods used to compare groups for primary and secondary outcomes
Descriptive statistics were calculated and Chi-square analyses were used to characterize the study groups at baseline and to determine the use of the website. We used paired t-tests to evaluate the differences at T1 and T2, for the intervention group and the control group. Subsequently we applied the method of General Linear Modeling with repeated measures to explore the overall change between the intervention and control group. Because we noticed from the process evaluation data that 16 healthcare professionals in the intervention group did not start using the website, we applied the method of General Linear Modeling with repeated measures again; for this analysis these 16 professionals were transferred to the waiting list control group. IBM Statistical Package for Social Sciences Inc. version 20.0 was applied and a p-value of ≤0.05 was considered as statistically significant.
12a-i) Imputation techniques to deal with attrition / missing values
no imputation techniques were used
12b) CONSORT: Methods for additional analyses, such as subgroup analyses and adjusted analyses
Because we noticed from the process evaluation data that 16 healthcare professionals in the intervention group did not start using the website, we applied the method of General Linear Modeling with repeated measures again; for this analysis these 16 professionals were transferred to the waiting list control group.

RESULTS

13a) CONSORT: For each group, the numbers of participants who were randomly assigned, received intended treatment, and were analysed for the primary outcome
A total of 69 healthcare professionals were identified, with 42 professionals participating in the intervention group and 27 in the control group at baseline (T1).
13b) CONSORT: For each group, losses and exclusions after randomisation, together with reasons
A total of 12 professionals were ‘lost’, 9 for not using their login code and 3 of them changed their email accounts.
13b-i) Attrition diagram
Figure 1. Flow chart intervention group and control group

14a) CONSORT: Dates defining the periods of recruitment and follow-up
Data collection via the website took place at T1 and T2 after 12 months. Initially the follow-up period was 6 months, however due to low participation rates, we extended the follow-up period. Professionals used the website from January 2011 till June 2012.
14a-i) Indicate if critical “secular events” fell into the study period
no critical events

14b) CONSORT: Why the trial ended or was stopped (early)
Data collection via the website took place at T1 and T2 after 12 months. Initially the follow-up period was 6 months, however due to low participation rates, we extended the follow-up period. Professionals used the website from January 2011 till June 2012.
15) CONSORT: A table showing baseline demographic and clinical characteristics for each group
Table 1 – Baseline demographics of study participants
N=69Intervention group (n=42)Control group (n=27)Sig.
Gender, female69.0%77.8%ns
Age, years38.6 (11.3)39.7 (8.4)ns
Education, bachelor degree78.8%68.4%ns
Education, degree above bachelor21.2%31.6%
Professional experience, years9.76 (8.5)9.58 (9.1)ns
Working as a soloist, or with 1 or 2 colleague's44.4%20.8%ns
Working with 3 or more colleague's55.6%79.2%
Consultation time devoted to health education59,2%54,4%ns
15-i) Report demographics associated with digital divide issues
Table 1 – Baseline demographics of study participants
N=69Intervention group (n=42)Control group (n=27)Sig.
Gender, female69.0%77.8%ns
Age, years38.6 (11.3)39.7 (8.4)ns
Education, bachelor degree78.8%68.4%ns
Education, degree above bachelor21.2%31.6%
Professional experience, years9.76 (8.5)9.58 (9.1)ns
Working as a soloist, or with 1 or 2 colleague's44.4%20.8%ns
Working with 3 or more colleague's55.6%79.2%
Consultation time devoted to health education59,2%54,4%ns
16a) CONSORT: For each group, number of participants (denominator) included in each analysis and whether the analysis was by original assigned groups
16-i) Report multiple “denominators” and provide definitions
N=69Intervention group (n=42)Control group (n=27)Sig.
Gender, female69.0%77.8%ns
16-ii) Primary analysis should be intent-to-treat
We used paired t-tests to evaluate the differences at T1 and T2, for the intervention group and the control group. Subsequently we applied the method of General Linear Modeling with repeated measures to explore the overall change between the intervention and control group. Because we noticed from the process evaluation data that 16 healthcare professionals in the intervention group did not start using the website, we applied the method of General Linear Modeling with repeated measures again; for this analysis these 16 professionals were transferred to the waiting list control group.
17a) CONSORT: For each primary and secondary outcome, results for each group, and the estimated effect size and its precision (such as 95% confidence interval)
Power analysis estimated how many respondents were needed for the study to find a significant difference in healthcare professionals' behavior. This analysis (power 0.80; alpha=0.05, two-tailed) revealed that 62 professionals in each condition were needed.
17a-i) Presentation of process outcomes such as metrics of use and intensity of use
Figure 2 provides a representation of the use of different modules on the website. The module to extend the professional patient-centered behavior to optimize processes of shared-decision making and self management, was used by 45.2% of the professionals (n=19). Of the professionals in the intervention group, 19.0% (n=8) used only one of seven screens; 7.1% (n=3) used all seven screens. The screen to support thinking about encouraging patients was used by 38.1% (n=16) of the professionals. In 19.0% (n=8) of the cases, pros and cons of encouraging patients in the short- and long-term were listed; 14.3% (n=6) used the screen to seek support, and 16.7% (n=7) looked at the sub-skills needed. In addition, the screen for planning the encouraging behavior was used by 16.7% (n=7) of the professionals; for putting the behavior change into practice by 9.5% (n=4); and, for maintaining the encouraging behavior 16.6% (n=7).
Figure 2. Labels

The module with background information on how to coach the patient with the purpose to support the health professional in his encouraging behavior towards patients was used by 47.6% (n=20) of the professionals; 16.7% (n=7) of the professionals used all the seven screens. 45.2% (n=19) used the screen how to encourage a patient to think about his/her personal risk; 33.3% (n=14) used the screen how to list pros and cons with a patient; 26.2% (n=11) used the screen how to seek support, and; 21.4% (n=9) how to practice the sub-skills needed. The last screens in this module (planning the encouraging behavior, putting the behavior change into practice and maintaining the behavior, were used by 19.0% (n=8) of the healthcare professionals. The forum directed at improving social support was used by 4 healthcare professionals.
Healthcare professionals invited 54 patients in the intervention (55.6% male, 50.9±11.8 years, differing educational degrees). Healthcare professionals assessed cardiovascular risk and 18.6% (n=10) of the patients had 2 or more cardiovascular risk factors, and/or a heart disease and/or diabetes; 13% (n=7) were physically active for at least 30 minutes, for 5 days per week. In 81.5% (n=44) of the patients, the guidelines to assess cardiovascular risk were not used by the health professional. The module to improve patients' intention and risk-reduction behavior with the purpose to increase the processes of shared-decision making and self management, was provided to 44.4% (n=24) of the patients by a health professional. Of the professionals that used this module together with their patients, 42.6% (n=23) provided their patient(s) with risk-perception. A 39.0% (n=21) provided attitudinal change and outcome expectations; 29.6% (n=16) resistance to social pressure and seek support, and 27.8% (n=15) provided encouraging sub-skill enactment. For 24.1% (n=13) of the patients the professional provided a planning of the behavior change; 18.5% (n=10) provided putting the behavior change into practice and 14.9% (n=8) provided maintaining the behavior change (Figure 2).

17b) CONSORT: For binary outcomes, presentation of both absolute and relative effect sizes is recommended
-
18) CONSORT: Results of any other analyses performed, including subgroup analyses and adjusted analyses, distinguishing pre-specified from exploratory
When we narrowed the intervention group (n=26) by transferring the professionals who did not use the website to the waiting list control group (n=43), no significant differences between the intervention and control group were found in social-cognitive determinants, intention and behavior, except for perceived behavior control and barriers (table 3). There was a difference in perceived behavioral control in the intervention group (t-test=-2.485, p=0.02, effect size=-0.30), and also in the control group (t-test=-3.105, p=0.00, effect size=-0.23). This detected difference in perceived behavioral control turned out to be overall non-significant. Results showed a significant overall difference in barriers between the intervention and the control group (F-test=4.128, p=0.02). Professionals in the intervention group experienced higher levels of barriers to encourage patients, than professionals in the control group.

18-i) Subgroup analysis of comparing only users

When we narrowed the intervention group (n=26) by transferring the professionals who did not use the website to the waiting list control group (n=43), no significant differences between the intervention and control group were found in social-cognitive determinants, intention and behavior, except for perceived behavior control and barriers (table 3). There was a difference in perceived behavioral control in the intervention group (t-test=-2.485, p=0.02, effect size=-0.30), and also in the control group (t-test=-3.105, p=0.00, effect size=-0.23). This detected difference in perceived behavioral control turned out to be overall non-significant. Results showed a significant overall difference in barriers between the intervention and the control group (F-test=4.128, p=0.02). Professionals in the intervention group experienced higher levels of barriers to encourage patients, than professionals in the control group.

19) CONSORT: All important harms or unintended effects in each group

-

19-i) Include privacy breaches, technical problems

-

19-ii) Include qualitative feedback from participants or observations from staff/researchers

-

DISCUSSION

20) CONSORT: Trial limitations, addressing sources of potential bias, imprecision, multiplicity of analyses

20-i) Typical limitations in ehealth trials

Limitations. In a review by Légare it was concluded that sufficient enrollment of healthcare professionals and patients is often a problem and needs attention in research designs [1]. Our estimated sample size was not achieved, although recruitment and follow-up period was extended, and strong attempts were made to encourage professionals. Changing intentions and the encouraging behavior of healthcare professionals and patients showed difficult with many inhibiting factors. An explanation is probably a ceiling effect for intention and attitude, so little progress can be expected in the intervention group. This combined with that our measures were based on self-reported intention and behavior that can have caused recall bias. Also, a possible explanation is a selection of professionals with special interest in health education, participating in our study. Although randomization made that participants were evenly distributed, we noticed that a few professionals were trained for another intervention but were not selected and they choose to attend our intervention. A limitation that might have influenced outcomes is that the website had to be applied during regular consultation with the patient. Though professionals stated that they spend much of their consultation time on health education, it showed not just a matter of focus on self management during consultation time. Another limitation that might influence the outcome is that the professionals needed more training how to work with the modules of the Web-based intervention.

21) CONSORT: Generalisability (external validity, applicability) of the trial findings

21-i) Generalizability to other populations

Since attendance and use of the Web-based intervention was sub-optimal, we cannot conclude that the intervention is ineffective; rather, the healthcare professionals were not able to extend their professional encouraging behavior. Results showed that healthcare professionals experienced higher levels of barriers meaning that time and organizational constraints withheld them and obstruct the planned behavior to encourage patients, compared with the professionals in the control group. Further research embedded in professionals' regular consultations with patients is required with specific emphasis on the processes of dissemination and implementation of innovations in patient-centered care.

21-ii) Discuss if there were elements in the RCT that would be different in a routine application setting

The setting was usual care

22) CONSORT: Interpretation consistent with results, balancing benefits and harms, and considering other relevant evidence

22-i) Restate study questions and summarize the answers suggested by the data, starting with primary outcomes and process outcomes (use)

The application of evidence-based behaviour change techniques used in our intervention should offer insight regarding how an intervention may change intention and behaviour. When intention and perceived behavioral control are targeted in an intervention, clinician behavior can be improved [34]. Methods used in our Web-based intervention were action planning and coping planning, however better results on intention and the (maintenance) of the behavior change were not reached [35]. Professionals' perception of perceived behavioral control is an important determinant of behavior to improve shared-decision making [1]. The use of the method guided practice with feedback did probably lead to increased perceived behavioral control and better skills. But, use of the method decisional balance to encourage listing of pros and cons of changing the behavior, did not lead to better results on attitudes. The use of the method resistance to social pressure and mobilizing others for social support, showed a slight but non-significant increase in subjective norm. We showed that professionals did not use or discontinue using the Web-based intervention. Though healthcare professionals stated that they spend a lot of time on health education (58,4% in the intervention group and 54.4% in the control group), and the pretest showed that working more evidence-based and systematic could be done in the same time, healthcare professionals lost interest in the intervention and stopped using the application.This non-use of the intervention or nonusage attrition is a documented problem in the search for intervention effects [13]. Eysenbach [13] called this the methodological challenges in the evaluation of eHealth applications. A clinical decision support system can improve healthcare professionals' performance when users are automatically prompted to use the website; but in our study the professionals themselves had to activate the system making [17]. A factor that may have influenced performance and attrition may be a lack of (immediate) advantages of working with the website for the healthcare professionals, or even encountering obstructions when working with the website. Another factor that may have influenced performance and attrition may be the compatibility with usual care and with the workload. And also the complexity of the intervention with (to) many modules may have influenced performance and attrition. The Web-based intervention was carefully developed following the Intervention Mapping protocol [7]. Healthcare professionals from the target group were involved in the development of the intervention, and they also pretested the application, but maybe (to) much attention was directed at 'does the website work as intended'.

22-ii) Highlight unanswered new questions, suggest future research

Results showed that healthcare professionals experienced higher levels of barriers meaning that time and organizational constraints withheld them and obstruct the planned behavior to encourage patients, compared with the professionals in the control group. Further research embedded in professionals' regular consultations with patients is required with specific emphasis on the processes of dissemination and implementation of innovations in patient-centered care. Training-on-the-job may improve eHealth use in healthcare settings.

Other information

23) CONSORT: Registration number and name of trial registry

Trial ID: ECP-92, NTR2584

24) CONSORT: Where the full trial protocol can be accessed, if available

Barbara.Sassen@hu.nl

25) CONSORT: Sources of funding and other support (such as supply of drugs), role of funders

none

X26-i) Comment on ethics committee approval

The study was approved by the Research Ethics Board at Maastricht University and was registered in the Dutch Trial Register (Trial ID: ECP-92, NTR2584).

x26-ii) Outline informed consent procedures

Healthcare professionals were the unit of randomization and were randomly allocated to the intervention vs. the waiting-list control group. Healthcare professionals invited their patients. The recommendation was to use the intervention for every patient that fit the intervention. Patients were informed about the study and gave informed consent.

X26-iii) Safety and security procedures

Usual care

X27-i) State the relation of the study team towards the system being evaluated

None declared
